# The Complications of Hemorrhoidectomy From Patients' Perspective: A Qualitative Study

**DOI:** 10.1002/hsr2.70724

**Published:** 2025-04-28

**Authors:** Masoumeh Ebrahimi Tavani, Yegane Partovi, Tahmineh Poursaki, Farid Gharibi

**Affiliations:** ^1^ Academic Research Staff, Quality Improvement, Monitoring and Evaluation Department, Center of Health Network Management, Deputy of Public Health Ministry of Health & Medical Education Tehran Iran; ^2^ Health Services Management, Department of Public Health, School of Public Health Ardabil University of Medical Sciences Ardabil Iran; ^3^ Department of Health Management, Policy and Economics, School of Public Health Tehran University of Medical Sciences Tehran Iran; ^4^ Health Services Management, Social Determinants of Health Research Center Semnan University of Medical Sciences Semnan Iran

**Keywords:** complication, hemorrhoidectomy, patient's perspective, qualitative research

## Abstract

**Background:**

Hemorrhoidectomy is a common general surgical procedure with numerous and intensive complications.

**Objective:**

This study sought to investigate the complications of hemorrhoidectomy from the patients' perspective.

**Methods:**

This qualitative study employed a phenomenological approach to explore the experiences of 27 patients with a history of hemorrhoidectomy. Convenience sampling was utilized initially, but purposive sampling was used as the investigation continued. All participants' statements were documented after obtaining informed consent and permission to record their experiences. Interviews were conducted until data saturation was achieved. The interview data was analyzed using content analysis, which involved the systematic extraction, interpretation, and reporting of the concepts and themes in the data.

**Results:**

The study identified three main categories of complications: physical, psychological, and social. Additionally, 26 themes and 56 sub‐themes were identified and defined under the main categories. Physical complications (PhCs) included nutritional problems, pain, sleep disturbances, physical weakness, bleeding, fecal incontinence, excretion problems, infection, fissures, anal prolapse, urinary retention, and disease recurrence. Psychological complications (PsCs) included fear, aversion, denial, isolation, depression, boredom, embarrassment, feeling defective, and dependency; Social complications (SCs) included secrecy, irritability, social withdrawal, and inability to perform social roles.

**Conclusions:**

In addition to common PhCs such as pain and bleeding, patients with a history of hemorrhoidectomy face numerous PsCs and SCs that need to be communicated to the patient before deciding on this surgery. Furthermore, doctors should also consider special measures to manage these complications properly.

## 1 Introduction

Hemorrhoidal disease (HD) is the most common condition affecting the rectum and large intestine, with a global prevalence estimated between 2.9% and 27.9% of the population [1, 2]. Studies indicate that the symptomatic type of this disease affects over 1 million people annually in Western countries [3]. For example, 39% of adults in Australia suffer from this disease, with half of them experiencing symptomatic forms [4]. Hemorrhoids, which account for over 70% of anorectal disorders, involve the swelling, congestion, and prolapse of the blood vessels in the anal wall. The primary cause of hemorrhoids is constipation in the gastrointestinal tract [5]. Hemorrhoids, in simple terms, are varicose veins located in the anorectal region [6]. Patient age distribution data indicate that the highest incidence of the disease occurs between the ages of 45 and 65, with a decline in incidence after this age [2, 7].

The treatment of HD disease depends on the progression and symptoms of the disease. Depending on the grade and severity, treatment can range from dietary recommendations, lifestyle modifications, and medication to rubber band ligation and surgery [8, 9]. Treatment options for symptomatic hemorrhoids are diverse and include conservative clinical treatments, nonsurgical methods, and various surgical procedures. Nonsurgical treatments for hemorrhoids include rubber band ligation, injection sclerotherapy, cryotherapy, infrared coagulation, and laser treatment, all of which can be performed without anesthesia. These methods are typically used as initial treatments and are often applied in the early stages of the disease [10].

Indications for surgical treatments include the presence of anal warts, hypertrophied papillae, fissures, severe thrombosis, and recurrence of symptoms after continuous use of rubber band ligation [11]. Hemorrhoidectomy is an effective surgical treatment for symptomatic grade three and four hemorrhoids and involves various methods, although open hemorrhoidectomy remains the most common procedure [12, 13, 14]. It is noteworthy that the severity and likelihood of complications from hemorrhoid surgery are such that many surgeons are reluctant to perform it and consider it a last resort [15]. Complications of hemorrhoid surgery can include pain, bleeding, involuntary defecation and flatulence, long‐term wound healing, defecation urgency, stricture, prolapse, external hemorrhoid thrombosis, pelvic infection, and peritonitis [16, 17, 18, 19]. Recurrence of the disease is also a significant complication. For instance, a study that followed up with surgically treated patients over 7 years found that 40% experienced a disease recurrence [20].

Severe pain following hemorrhoid surgery is the most common issue observed in all patients. In addition to other short‐term complications, urinary retention is observed in 20.1% of cases, hemorrhage in 2.4%–6%, and subcutaneous abscess in 0.5% of cases. Long‐term complications occurred in 2.6%–51% of cases, with anal fissures, anal strictures, anal incontinence, and fistulas occurring in 1%, 0.4%, and 0.5% of cases, respectively [21, 22]. The anal canal tissue is the richest in nerves within the gastrointestinal tract, so pain after hemorrhoid surgery, especially during bowel movements, is natural [23, 24].

Recent studies on HD have predominantly focused on its pathological, epidemiological, and therapeutic aspects but have not deeply examined the experiences of surgically treated patients [14]. Although the review of studies conducted on common complications of hemorrhoidectomy surgery has led to identifying a range of PhCs, the complications identified in different studies are not exactly similar, which could be due to incomplete investigation of the subject. These severe and long‐term PhCs identified will have a significant and profound effect on the patient's psyche and social roles, an issue that has been neglected by researchers and even therapists so far. Therefore, conducting a comprehensive study with a qualitative approach to identify all PhCs of hemorrhoidectomy surgery and also to identify the possible PsCs and SCs of the surgery on the patient will be very necessary. Therefore, this study sought to investigate all of the complications of hemorrhoidectomy from the patients' perspective.

## 2 Methods

### 2.1 Study Type

This qualitative study, conducted in 2024, employed a phenomenological approach. Phenomenology is a type of qualitative research scrutinizing the experiences of individuals who have encountered an unknown phenomenon.

### 2.2 Participants

The study included 27 patients who had undergone hemorrhoidectomy at Kosar Hospital in Semnan, Iran, with at least 1 year passed since their surgery. The study participants consisted of patients with various types of hemorrhoids (internal, external, prolapsed, and thrombotic) who were forced to undergo hemorrhoidectomy surgery due to disease progression and reaching stages 3 and 4 of the disease and experiencing severe bleeding and pain. Patients' data was acquired from the hospital's medical records department, and all patients received phone calls encouraging them to participate in the research. Initial sampling was done on an available basis, but purposive sampling was used as the study progressed. This means the focus was on individuals who could provide the researchers with the most comprehensive information [25]. Purposive sampling allows the researcher to extract and report conceptual patterns related to the nature of events in the minds of individuals in the best possible way [26].

### 2.3 Study Tool and Data Collection

In this study, researchers used structured interviews to examine participants' experiences [27]. The interview checklist (Appendix 1) included these questions: “Please explain your experience with HD and the surgery you had, and what has happened to you since then?”; “What PhCs did you experience after surgery?”; “What PsCs did you experience after the surgery?”; “What SCs did you experience after the surgery? Were your social roles disrupted?”. The interview checklist was developed after consulting seven specialists in colorectal surgery, general surgery, physiology, and qualitative methodology in nursing and health services management. Individual interviews were conducted with each study participant, lasting between 25 and 70 min. Interviews were conducted after obtaining informed consent and permission to record the participants' conversations. Interviews continued until data saturation.

### 2.4 Data Analysis

Content analysis was used to analyze the data obtained from the interviews, systematically extracting, interpreting, and reporting the concepts and themes in the statements [28]. The participants' statements were recorded on paper and repeatedly reviewed during transcription to ensure accuracy. In the subsequent coding phase, the experiences expressed by the participants were presented as codes. Similar codes were grouped to form sub‐themes based on the explicit or implicit themes in the codes. Finally, by merging related sub‐themes, primary or final themes were generated and identified, and the main themes were combined to form categories.

### 2.5 Study Rigor

The results obtained from the interviews were provided to the participants for validation to increase the study's rigor. Additionally, two researchers separately analyzed the data, and an external researcher compared their analyses. The researchers reached an agreement on the final findings through discussion. The checklist used to guide the implementation and reporting of results, as well as the critical appraisal of the present paper, is the CASP (Critical Appraisal Checklists) Qualitative Studies Checklist, which is consistent with the qualitative nature of this study.

### 2.6 Ethical Considerations

Several measures were taken to adhere to ethical principles, including the approval of the research proposal by the Ethics Committee (IR.SEMUMS.REC.1403.062), obtaining informed consent from study participants, informing participants of their right to freely participate in the interviews before the sessions, obtaining permission to record the interviews, and ensuring confidentiality and nondisclosure of interviewee identities.

## 3 Results

The average age of the patients in this study was 42 (±13) years, and 70.37% were men. Of course, the prevalence of the disease in Iran is almost equal in men and women. However, due to cultural restrictions, women's participation and cooperation levels were lower than men's. About 92.60% of the patients were married, all were literate, and 77.78% had an academic education. About 62.97% of the patients were employed, and 74% were urban dwellers. All participants had basic health insurance, and more than 70% had basic social security insurance, but only one‐third had supplemental health insurance. Participants' mean elapsed time from onset of symptoms to diagnosis was 6.8 years, while the mean elapsed time from diagnosis to start of care was 1.2 years. Also, about 60% of patients received required care from government and private centers (Table 1).

**Table 1:** Demographic and background characteristics of patients.

Demographic characteristics	Grouping	Frequency	Percentage
Age	Less than 30 years	2	7.41
	30–39 years	8	29.62
	40–49 years	11	40.74
	50 years and older	6	22.22
Gender	Female	8	29.63
	Man	19	70.37
Marital status	Married	25	92.60
	Separated	1	3.70
	Deceased wife	1	3.70
Educational level	High school	2	7.40
	Diploma	4	14.82
	Bachelor	18	66.67
	Masters	3	11.11
Employment status	Employee	6	22.22
	Manual worker	2	7.41
	Self‐employed	7	25.93
	Retired	2	7.41
	Homemaker	9	33.33
	Unemployed	1	3.70
Urban/rural	Urban	20	74.07
	Rural	7	25.93
Having basic insurance	Yes	27	100
	No	0	0
Type of basic insurance	Social Security	19	70.37
	Health Services	8	29.63
Having supplemental Insurance	Yes	9	33.33
	No	18	66.67
Time elapsed from onset of symptoms to diagnosis	1–4 years	10	37.04
	5–9 years	14	51.85
	Ten years or more	3	11.11
Time elapsed from diagnosis to start of care	Immediately	21	77.78
	Less than one year	4	14.81
	1–4 years	2	7.41
Place of receiving care	Public centers	6	22.22
	Private centers	5	18.52
	Both public and private centers	16	59.26

The study identified three main categories of complications: physical, psychological, and social. Additionally, 26 themes and 56 sub‐themes were identified and defined under the main categories (Table 2).

**Table 2:** Identified complications in patients with a history of hemorrhoid surgery.

Physical Complications (PhCs)	Nutritional problems	Severe loss of appetite and poor eating habits
		Refusal to eat due to fear of going to the bathroom
		Stomach discomfort and indigestion
	Pain	Severe burning sensation in the anal area
		Persistent, severe, and prolonged anal itching
		Feeling of heaviness and intense pressure in the anal and buttocks area
		Severe pain in the anus and buttocks while sitting
		Excruciating pain during bowel movements when stool is hard
		Pain and pressure in the anus due to gas retention and inability to pass it
		Severe pain during physical activity
	Sleep problems	Prolonged insomnia due to severe pain
		Waking up due to anal pressure and the urge to defecate
	Physical weakness	Severe weakness, dizziness, blurred vision, extreme fatigue
		Weight loss and muscle mass reduction
	Bleeding	Severe bleeding during defecation, especially with hard stools
		Involuntary discharge of blood and other secretions
		Anemia
	Loss of bowel control	Fecal incontinence
		Inability to control passing gas
	Defecation problems	Inability to defecate properly
		Inability to pass intestinal gas, severe pain
		False sensation of needing to defecate
		Prolonged defecation process, increasing pain
	Infection	Infection at the wound site
		Inflammation of the anus and surgical site
		Development of fistulas and abscesses
	Fissure	Development of fissures at the surgical site
	Rectal prolapse	Prolapse or protrusion of the rectum
	Constipation	Frequent constipation
	Urinary retention	Inability to fully empty the bladder
	Recurrence of disease	Recurrence of the disease and symptoms
Psychological Complications (PsCs)	Fear	Fear of going to the bathroom and eating
		Fear of catching a cold or allergies due to sneezing
		Fear of prolonged defecation
		Fear of wound infection
		Fear of disease recurrence
		Fear of repeated surgery
		Fear of constipation
		Fear of frequent and close bowel movements
	Aversion	Disgust towards foods consumed post‐surgery
		Disgust towards individuals and things reminding of the post‐surgery period
	Denial	Denial of bowel movements, attributing them to false sensations
		Denial of disease recurrence and need for additional surgery
	Social withdrawal and isolation	Lack of desire to interact with others
	Depression	Feeling hopeless and helpless
	Boredom and Frustration	Lack of motivation and mental confusion
	Embarrassment	Feeling ashamed and embarrassed
	Feeling defective	Feeling incomplete and defective
	Dependency	Dependence on medications
Social Complications (SCs)	Secrecy	Hiding surgery from others
		Concealing the reason for the surgery
	Irritability	Unnecessary conflicts with family members due to pain, lack of sleep, and stress
	Avoidance of social gatherings	Reluctance to be in groups
	Inability to fulfill social roles	Inability to perform personal and family duties
		Inability to fulfill marital responsibilities
		Inability to perform job duties

**■ Physical Complications (PhCs)**

The most common PhCs and problems for patients are as follows:

**Nutritional problems**

– ***Severe loss of appetite and malnutrition***
  A noticeable decrease in appetite for various reasons, such as severe anemia due to multiple bleeding, recurring pain, and even fear of eating due to concerns about excretion, are among the complications of hemorrhoid surgery.
  “Due to prolonged periods of not eating, especially during colonoscopy and surgery, and also restrictions on eating afterward, my appetite gradually decreased.”
  (Participant 3)
– ***Refusal to eat due to fear of going to the bathroom***
  Thinking about the end of eating leading to going to the bathroom causes patients who have undergone surgery to refrain from eating or consuming minimal amounts of food. However, this very issue will also delay the recovery of patients.
  “When I thought about going to the bathroom, I lost my motivation to eat.”
  (Participant 12)
– ***Stomach discomfort and indigestion***
  Continued insufficient food intake leads to various problems, particularly stomach and intestinal ulcers. This is because a portion of stomach acid secretion is continuous and unrelated to food intake. When the stomach remains empty of food, stomach acid can damage the stomach lining, resulting in pain and ulcers.
  “I felt a severe burning sensation in the area below the chest and had to take medications to reduce stomach acid, neutralize it, and also heal the stomach ulcer.”
  (Participant 20)
**Pain**
– ***Intense burning sensation in the anal area***
  A sensation of severe and continuous pain is one of the main characteristics of hemorrhoid surgery, often manifesting as severe burning or intense spasms.
  “I have experienced severe pain throughout my life due to illnesses, fractures, and even numerous surgeries, but I have never experienced pain like this before.”
  (Participant 2)
– ***Persistent, severe, and long‐term itching in the anal area***
  Another PhCs of hemorrhoid surgery is intense itching in the anal area, especially noticeable several weeks after the surgery, and can persist for months.
  “The itching intensity was such that sometimes I was compelled to scratch my anus in front of others, which would draw disapproving stares and subsequently lead to embarrassment and a loss of dignity for me.”
  (Participant 15)
– ***The feeling of heaviness and intense pressure in the anal and pelvic area***
  Another consequence of hemorrhoid surgery is a sensation of heaviness in the anal and pelvic area, sometimes accompanied by severe and painful contractions at the end of the intestine. This heaviness often manifests as a false urge to defecate and can be due to inflammation or severe tissue damage in the large intestine.
  “I experienced a heavy feeling and severe contractions in my pelvis. Many times, I felt a false urge to pass gas or stool, prompting me to urgently rush to the toilet, only to find no relief.”
  (Participant 15)
– ***Severe pain in the rectum and pelvic area when sitting***
  Severe pain when sitting is a common observation in individuals who have undergone hemorrhoid surgery. This can be attributed to the surgery's intensity, the operated area's sensitivity due to a high concentration of nerves, and the significant pressure exerted on the surgical site during sitting.
  “The excruciating and unbearable pain, especially when sitting, is the hallmark of this surgery.”
  (Participant 24)
– ***Severe and exhausting pain during hard stool passage***
  Severe pain during the first bowel movement after surgery is widely reported among patients. Initial bowel movements are typically soft and watery due to the foods consumed, but as stools become firmer and more solid over time, the pain intensifies.
  “As my stool became firmer over time, the pain during passing and even continuous contractions afterward increased.”
  (Participant 12)
– ***Pain and pressure in the rectum due to gas retention and inability to expel it***
  Feeling severe pain and pressure in the pelvis, especially around the surgical area, isn't limited to passing stool alone. Patients also endure exhausting pain when trying to pass intestinal gas. The major post‐surgery discomfort and issues stem from the fact that this condition affects an organ that cannot remain inactive, unlike a broken hand or leg.
  “I faced severe problems trying to pass intestinal gas because it was very difficult to release it, and I experienced severe pain during this process.”
  (Participant 4)
– ***Severe pain during physical movement***
  Since the middle part of the body is the center of gravity during physical activities and physical pressure, any bodily movement, such as walking, lifting objects, and even sitting or bending down, leads to severe pain in the pelvis and the surgical area.
  “For months after the surgery, I couldn't engage in physical activities because I experienced intense pain and even bleeding.”
  (Participant 12)
**Sleep problems**
– ***Long‐term insomnia due to severe pain***
  Insomnia is a significant side effect of hemorrhoid surgery. Patients often require adequate sleep due to physical and psychological conditions, but they cannot sleep properly due to exhausting physical pain.
  “One of the biggest problems I faced was insomnia because severe pain often prevented me from sleeping.”
  (Participant 25)
– ***Waking up from rectal pressure from urgency***
  Patients who manage to sleep despite severe pain often cannot have sufficient, complete, and uninterrupted sleep due to issues such as intense and sometimes false sensations of urgency, burning, and severe contractions in the intestines. Many patients resort to medications like diazepam and alprazolam (and even pain relievers like tramadol) to alleviate sleep problems.
  “If I managed to fall asleep due to exhaustion or taking pain relievers or sleep aids, I would often wake up shortly afterward due to pain, itching, burning, or intense pressure from gas or the urge to defecate.”
  (Participant 1)
**Physical weakness**
– ***Severe weakness, dizziness, visual blackouts, and excessive fatigue***
  Due to post‐surgery pain, loss of appetite, insomnia, and lack of mobility, patients gradually develop severe physical weakness, making it difficult for them to perform even the simplest tasks.
  “I experienced a darkened vision, intense weakness, and dizziness in my hands and feet for about 3 months.”
  (Participant 25)
– ***Weight loss and muscle mass reduction***
  It's common for patients to experience weight loss and reduction in muscle mass following hemorrhoid surgery, primarily due to various factors such as poor nutrition and prolonged loss of appetite. This can lead to severe weakening and decreased physical capacity in the patient.
  “During my recovery period, I lost about 12 kilograms, with a significant portion of it occurring in the first few months after the illness.”
  (Participant 10)
**Bleeding**
– ***Severe bleeding during defecation, especially when the stool is firmer***
  Extensive tissue damage in the large intestine and rectum can lead to ongoing bleeding for weeks. This bleeding intensifies significantly during firmer bowel movements and can be quite substantial.
  “For several weeks after the surgery, I experienced bleeding every time I had a bowel movement, especially when the stool was firmer.”
  (Participant 8)
– ***Involuntary leakage of blood and various discharges normally***
  The involuntary leakage of blood and other discharges from the rectum is not limited to bowel movements but can persist unintentionally for weeks, whether during sleep or wakefulness.
  “I noticed bleeding on numerous occasions, most notably during bowel movements, but it often occurred unexpectedly in everyday situations.”
  (Participant 17)
– ***Anemia***
  Anemia can develop due to repeated bleeding during bowel movements or intermittently, as well as due to nutritional deficiencies and inadequate iron intake in patients.
  “I was fragile and pale, constantly feeling dizzy and experiencing blurred vision. After some time, I went for a check‐up and found out I had severe anemia.”
  (Participant 20)
**Loss of bowel control**
– ***Fecal incontinence***
  Involuntary bowel movements are a serious issue faced by post‐surgery patients, occurring due to extensive tissue damage in the large intestine and rectum and the individual's inability to control their anal sphincters. This problem can persist for several weeks.
  “For several months, I experienced involuntary bowel movements. This happened unpredictably and uncontrollably everywhere.”
  (Participant 6)
– ***Involuntary gas release control***
  Loss of control over passing gas is not limited to bowel movements; involuntary gas release can occur more frequently and intensively than stool. Typically, gaining control over bowel gas expulsion can take much longer than controlling bowel movements, and patients facing this problem often experience severe social anxiety and insecurity as a result.
  “I still struggle with issues like loss of control over the gas release, especially at night or when it's been several hours since I last went to the bathroom.”
  (Participant 6)
**Difficulty with defecation**
– ***Inability to defecate properly***
  Despite having limited control over the rectum to retain small, soft stools, patients face significant challenges in regular or firm stool defecation, especially in the first few weeks after surgery. The reason for this lies in tissue destruction, the presence of wounds, stitches, and inflammation in the anal and rectal area.
  “For weeks after surgery, I couldn't pass stools properly. It felt like the passage for stool through my rectum and anus was blocked. I would sit in warm baths to try to have some bowel movements.”
  (Participant 11)
– ***Inability to expel intestinal gas and severe pain***
  Although patients who have undergone surgery can partially control intestinal gas (typically at least 1 month after surgery), in the first few weeks after surgery, expelling gas from the stomach can be a severe problem, often accompanied by pain.
  “During the first few weeks after surgery, I had trouble expelling intestinal gas. It felt like my intestines and rectum were completely closed due to inflammation from the surgical tissues.”
  (Participant 18)
– ***False sensation of defecation***
  Experiencing a false sensation of needing to defecate is a common complaint among patients. This issue can arise due to inflammation in the intestines and surgical tissues, which create a sense of fullness and pressure that triggers pain receptors.
  “Several times a day, I would need to go urgently to the bathroom, but there was nothing to pass.”
  (Participant 18)
– ***Prolonged defecation process and increased pain***
  A quick and complete bowel movement can benefit patients by reducing localized pressure on the surgical area. However, due to the extent and depth of the surgical site, many patients cannot achieve this, leading to a prolonged defecation process that increases pressure and pain.
  “I couldn't defecate quickly and all at once, which would have relieved a lot of pressure and pain.”
  (Participant 9)
– ***Infection at the surgical wound site***
  One possible complication following extensive and sensitive surgeries, such as those involving the intestines, is infection at the surgical wound site. Despite using strong antibiotics like gentamicin post‐surgery and sitting in a basin containing povidone‐iodine solution several times a day, infections at the wound site can still occur.
  “Once, my pain became significantly worse than usual, and at the same time, there was foul‐smelling discharge and blood from my rectum. Unfortunately, my wound had become infected.”
  (Participant 5)
– ***Inflammation at the rectum and surgical site***
  One of the common complications of hemorrhoid surgery is inflammation at the surgical site and rectum, which can persist for months. This issue can arise due to the extent and intensity of the surgery and the sensitivity of the affected area.
  “I experienced severe inflammation at the end of the intestine, rectum, and even the surrounding external tissues.”
  (Participant 12)
– ***Development of fistula and abscess***
  It is quite probable for patients who have undergone hemorrhoid surgery to encounter complications such as fistulas or abscesses. These conditions often result from infection, requiring further hospitalization and sometimes additional surgeries. They are considered among the worst complications of hemorrhoid surgery.
  “About 2 months after surgery, I developed an infected abscess. After some time, I had to undergo surgery again, leading to new challenges for me.”
  (Participant 24)
– ***Development of anal fissure***
  Anal fissure refers to an internal tear in the tissue lining of the anus, characterized by severe burning pain and bleeding during defecation. It often occurs due to straining and pressure, especially during constipation.
  “During defecation, I experienced severe bleeding and intense pain.”
  (Participant 11)
**Rectal prolapse**
– ***Rectal prolapse, or protrusion of the rectum***
  This condition occurs when pressure, particularly during bowel movements, causes a portion of the rectum to protrude. This condition is unpleasant for many patients, and its resolution often takes a long time.
  “… After passing stool, I felt like my rectum didn't return to its normal state. No matter how hard I tried manually, I couldn't get it back to normal. This condition caused pain and discomfort, which persisted for months.”
  (Participant 2)
**Constipation**
– ***Frequent episodes of constipation***
  This disorder can result from poor appetite, inadequate food intake, or consumption of improper foods lacking sufficient fiber. This can lead to insufficient food volume, subsequent lack of bowel distension to stimulate peristalsis, and difficulty passing stool. Many complications of the disease, such as severe pain, intense bleeding, development of fissures, or disease recurrence, are closely related to the occurrence of constipation.
  “During my recovery period, I endured severe pain and intense bleeding, feeling like the ulcers inside my intestine and rectum had freshly worsened.”
  (Participant 2)
**Urinary retention**
– ***Inability to empty the bladder completely***
  Despite the apparent separation of the digestive and urinary systems, they can impact each other due to their physical proximity. One complication of hemorrhoid surgery is urinary retention, where a person is unable to empty their bladder after visiting the toilet.
  “Due to this problem, I constantly felt the urge to urinate and experienced discomfort and even significant pain in my bladder.”
  (Participant 7)
**Relapse of the disease**
– ***Recurrence of the disease and symptoms***
  Despite all the measures taken by doctors, there is a possibility of the disease (hemorrhoids) recurring. For some individuals, the disease may recur in the first few months after surgery, while for others, it may happen in the subsequent years. In addition to the quality of the surgery performed, changes in the patient's lifestyle (especially diet and physical activity) significantly impact the recurrence of the disease.
  “I suffered a lot until I improved slightly, but after a while, about 2 months later, the disease recurred, and masses protruded again during bowel movements.”
  (Participant 2)
**■ Psychological Complications (PsCs)**
  Psychological problems arising from hemorrhoid surgery are very common. Here, we will discuss some of these issues.
**Fear**
– ***Fear of using the restroom and eating***
  Significant physical effects, such as pain and bleeding that occur after surgery can create anxiety about going to the restroom. This fear is not limited to going to the bathroom but also extends to eating because bowel movements result from eating.
  “In the first defecation after surgery, I suffered greatly; I was in severe pain in the bathroom and sweating profusely. This caused me to have anxiety about going to the bathroom; I even refrained from eating because I thought about defecation.”
  (Participant 11)
– ***Fear of catching a cold or allergies due to the possibility of sneezing***
  Sneezing exerts significant physical pressure on the body and causes pain in the intestines and anus of the patient. Since sneezing typically occurs during a cold or seasonal allergy flare‐up, it is understandable that patients fear catching a cold or allergies.
  “Every time I sneezed, I felt a lot of pain in the buttocks and pelvis area, especially in the anus. I was always worried about catching a cold, so I tried to dress warmly, even in warm weather.”
  (Participant 12)
– ***Fear of prolonged defecation***
  Prolonged bowel movements (and the inability to evacuate quickly) lead to increased pressure on the rectal area and can cause severe pain. For this reason, fear of developing constipation and delayed bowel movements is common among patients.
  “I was always afraid of prolonged defecation because it increased my pain.”
  (Participant 23)
– ***Fear of wound infection***
  Given the severe implications of wound infection following hemorrhoid surgery, patients are expected to fear encountering such infections. Surgical site infections prolong the treatment process, increase its cost, and can reduce the effectiveness of the surgery.
  “Hemorrhoids have frequently occurred in our family. That's why I always feared developing an infection.”
  (Participant 24)
– ***Fear of disease recurrence***
  Recurrence of hemorrhoids is observed in a significant portion of patients, leading them to worry about disease relapse. Proper postoperative care and lifestyle changes that contribute to initial hemorrhoids can reduce the likelihood of recurrence.
  “Having read online that disease recurrence is possible even after surgery, I was always anxious about this because I knew I couldn't endure such severe pain again.”
  (Participant 12)
– ***Fear of undergoing surgery again***
  The intensity of the side effects of hemorrhoid surgery is such that patients are always wary of undergoing surgery again. If these multiple fears are not appropriately managed, they can lead to severe psychological distress and potentially disease recurrence.
  “For months, I endured pain, bleeding, social isolation, and even depression during the recovery period. I am not willing to undergo this surgery again.”
  (Participant 14)
– ***Fear of constipation***
  Constipation leads to hard, painful bowel movements, often accompanied by rectal bleeding. Therefore, it is expected that patients would fear encountering constipation. While stress, if controlled and in moderation, might help in adhering to medical advice, excessive and uncontrolled stress will certainly be detrimental.
  “I always feared constipation, so I tried to eat high‐fiber foods like fruits and vegetables.”
  (Participant 8)
– ***Fear of frequent and close bowel movements***
  In addition to causing constipation and long‐lasting bowel movements, frequent and closely spaced bowel movements also lead to pain, mainly cramping in the pelvic and rectal areas. Hence, it is expected that patients would fear encountering this situation.
  “I was always anxious about frequent visits to the bathroom because they caused intense pressure and severe pain in the surgical area.”
  (Participant 8)
**Aversion**
– ***Aversion to foods consumed during the post‐surgery period***
  Given that hemorrhoid surgery and its recovery period are physically demanding for patients, it is expected that recalling this period or any related mention thereof can evoke unpleasant memories and sometimes distress.
  “The doctor recommended consuming plain lentil soup and drinking plenty of pineapple juice. I have intensely disliked their taste, smell, and even their name.”
  (Participant 12)
– ***Aversion to individuals and things reminiscent of the post‐surgery period***
  Environments and individuals can become triggers for the aversion created in patients. These triggers include clinics, diagnostic laboratories, and hospitals where the surgery was performed.
  “I felt repulsion towards anything that reminded me of the period of illness and recovery after surgery. I have been through so much hardship, and I don't think this aversion will ever disappear.”
  (Participant 20)
**Denial**
– ***Denial of defecation and associating it with false sensations of defecation***
  Pain and bleeding are prominent issues in this condition, especially when stool is passed. Therefore, it is natural for patients to deny the sensation of needing to defecate and attribute it to false urges.
  “I often denied many realities I felt; I even rejected the urge to defecate because I knew it would cause pain.”
  (Participant 1)
– ***Denial of disease recurrence and the need for re‐surgery***
  Denial of disease recurrence and the need for further surgery may perhaps be the greatest form of denial (and distortion of reality). Accepting this reality would mean facing the possibility of reliving all the hardships endured previously (potentially with greater intensity due to surgery in a previously operated area).
  “Despite knowing that my condition has returned and that I need another surgery, I try to deny this reality because accepting it would mean reliving all these events again.”
  (Participant 7)
**Social avoidance and isolation**
– ***Reluctance to engage with others***
  The psychological state of a post‐surgery patient is such that due to severe pain and ongoing challenges, they prefer to distance themselves from people. Patients often choose to cope with the consequences of their illness in solitude, away from everyone, ensuring that no one witnesses their uncomfortable condition.
  “During the recovery period after surgery, I didn't have a good emotional state and felt isolated. I avoided my children entering my room; I didn't want anyone to see me in that condition.”
  (Participant 12)
**Depression**
– ***The feeling of hopelessness and despair***
  Facing severe and ongoing consequences of illness and choosing to distance oneself from others can lead to depression in patients. Many patients reported experiencing various degrees and forms of depression during their recovery period.
  “The severe pain and other problems caused a state of depression in me. I cried often due to the complicated situation I found myself in.”
  (Participant 7)
**Boredom and fatigue**
– ***Lack of motivation and mental confusion***
  Insomnia and enduring multiple and continuous pains reduce the mental capacity and physical strength of post‐surgery patients. This condition, which can last for months, causes frustration in the patient and exhaustion in those around them.
  “I had no patience for anything or anyone. My tolerance for suffering had reached its breaking point, and I felt like a cup that had overflowed.”
  (Participant 5)
**Embarrassment**
– ***Embarrassment and shame***
  Due to the nature of the disease, the patient's privacy is frequently violated during the treatment procedure. Family members also experience unprecedented situations and conditions with the patient that are unpleasant for them. Therefore, it is natural for the patient to feel embarrassed by the medical staff, family members, and visitors.
  “This disease has made me embarrassed; the patient's sense of embarrassment is unimaginable because private parts of the body are repeatedly touched by medical staff.”
  (Participant 26)
**Feelings of defectiveness**
– ***Perceived inadequacy***
  A perception of inadequacy and defectiveness is a typical psychological condition among patients, resulting from factors such as lack of control over bowel movements, anatomical changes in the rectum, disruption of mental balance, and even forced limited relationships with others.
  “I used to be a very healthy person before contracting this disease. I felt like I would never be that person again.”
  (Participant 21)
**Dependency**
– ***Psychological dependence on drugs***
  Severe pain and insomnia during the recovery period can lead many patients to rely heavily on painkillers or strong sleeping medications. Pain management is a patient's right, but prescribing and using medications correctly to avoid creating addiction and dependence is crucial.
  “During my recovery period, due to intense pain, the doctor prescribed strong painkillers like tramadol for me. Because of the relief it provided, I was strongly inclined to use it, and even after recovery, I used it several times.”
  (Participant 18)
**■ Social Complications (SCs)**
  A neglected aspect of hemorrhoid surgery is the social problems it may cause. These consequences relate to disruptions in a person's role within their family, workplace, and social environment, which are discussed further below.
**Secrecy**
– ***Hiding surgery from others***
  Contracting this disease due to its extensive and long‐term implications can be considered a social stigma. This issue is especially significant for young individuals and unmarried women. Therefore, it is natural for patients to want to conceal their illness and even their surgery from others.
  “I am a single woman and feel that getting sick and undergoing surgery could affect the mindset of my peers and my marriage prospects.”
  (Participant 12)
– ***Secrecy regarding the reason for surgery***
  Because hemorrhoids and their treatment can cause discomfort to sensitive areas of the body, it's natural that people would want to keep their diagnosis and surgical history of hemorrhoids a secret.
  “I thought that if I told others that I had hemorrhoid surgery, it would lead to a loss of dignity and a weakening of my social standing.”
  (Participant 10)
**Irritability**
– ***Unnecessary conflicts with family members due to pain, insomnia, and psychological pressure***
  Pain, bleeding, insomnia, and severe and prolonged isolation can reduce the emotional capacity and resilience of a post‐surgery patient, severely impacting their ability to maintain proper relationships with others.
  “I would often pick fights over trivial matters and argue with my family.”
  (Participant 4)
**Social avoidance**
– ***Reluctance to join gatherings***
  For some patients, the inability to control bowel movements and stomach gas is so severe that they are unwilling even to attend their family gatherings. Other reasons for this may include the patient's physical weakness and inability to sit comfortably.
  “I didn't want anyone at home or anyone to visit me. This condition significantly affects the patient's self‐esteem and social standing.”
  (Participant 3)
**Inability to perform social roles**
– ***Inability to carry out family and personal tasks***
  Physical incapacity, severe pain when sitting or walking, lack of sufficient balance while walking, and other factors contribute to a person's inability to perform tasks. During this period, individuals rely heavily on their family, especially their spouse, who has been instructed for this care.
  “I was severely hindered in performing my tasks. I even lacked the ability and balance to bathe myself.”
  (Participant 11)
– ***Inability to fulfill marital duties***
  Physical incapacity in performing adequate movement, lack of emotional and psychological balance, as well as localized pain and contractions contribute to a person's inability after surgery or a lack of desire to engage in sexual relations. If attempted, significant pain, especially during climax, may be experienced.
  “For two to 3 months, I couldn't engage in sexual relations because of weakness and severe pain. Contractions in the genital and buttock areas during intercourse hindered this, causing intense pain.”
  (Participant 16)
– ***Inability to perform job duties***

Difficulty in fulfilling job responsibilities due to severe physical and psychological issues is a common issue among post‐surgery patients. This problem is severe for those with physically demanding jobs. Additionally, the inability to control bowel movements exacerbates this issue.

“I was on the verge of being fired, but several people intervened to prevent it. I worked in a factory, which required significant physical capability.”

(Participant 22)

## 4 Discussion

The results showed that the complications of hemorrhoidectomy can be categorized into three categories: physical, psychological, and social. PhCs identified in the study include nutritional problems, pain, sleep disturbances, physical weakness, bleeding, loss of bowel control, defecation issues, infection, formation of fissures at the surgical site, rectal prolapse, urinary retention, and recurrence of the disease.

Ielpo et al. (2010) aimed to examine early and late complications of hemorrhoidectomy; severe pain, urinary retention, hemorrhoid thrombosis, bleeding, and anal fissure were identified as major surgical complications [29]. A study by Harvitkar et al. in 2021, focusing on the treatment of hemorrhoids using laser therapy, reported pain, bleeding, urinary retention, abscess formation, stool incontinence, fistula formation, and stenosis as common side effects of hemorrhoid surgery [30]. Nikooiyan et al., in their 2016 study comparing the efficacy of surgical and electrotherapy treatments for hemorrhoids, identified main surgical complications such as inability to control gas in the rectum, severe pain, constipation, bleeding, urinary retention, and rectal prolapse [31]. Furthermore, a study by Keshkar et al. in 2011 investigating short‐term complications of hemorrhoidectomy in patients listed severe pain, bleeding, infection, inflammation, and inability to control bowel movements and gas as principal side effects [17].

The psychological effects of the disease include fears, aversion, denial, social withdrawal, depression, boredom, embarrassment, feeling defective, and developing dependence on painkillers and sleeping pills. The social consequences of the disease include concealing surgery, irritability, social avoidance, and inability to perform social roles. It's worth noting that no studies have yet specifically examined the psychological and social consequences of hemorrhoid surgery. Therefore, there is currently no basis for comparing the results of such a study with existing research.

Based on the results, the following recommendations can be proposed:

- **Nutritional Plan**: Provided by nutrition experts to ensure proper intake of micro and macronutrients, prevent constipation, and reduce bloating.
- **Medication Supplements**: Prescribed to prevent loss of appetite, weakness, anemia, and constipation.
- **Pain and Sleep Management**: Proper management to help patients through the recovery period without developing a dependency using effective medications [32]
- **Physiotherapy and Occupational Therapy**: Treatments such as biofeedback to help patients gain better and faster control over the rectum.
- **Laxatives**: The patient is prescribed these, along with dietary and exercise recommendations, to regulate the bowel movement process.
- **Effective Antibiotics**: Provided to prevent infections.
- **Hygiene Recommendations**: Use Betadine baths to avoid wound infections and teach the patient proper bowel movement techniques while emphasizing avoiding straining during defecation.
- **Psychological Counseling**: Provided to the patient with severe PsCs being examined by a psychiatrist and necessary medications prescribed.
- **Family Education**: Informing family members and other close individuals about the patient's condition.
- **Avoiding Dependency**: Preventing any physical and psychological dependency on various medications.
- **Employer Education**: Social workers from the hospital where the surgery occurred should inform the patient's employers.
- **Job and Insurance Support**: Providing job and insurance support to the operated patient.
- **Marital Counseling**: Provided to the patient and their spouse by an experienced psychologist familiar with the subject to improve conditions and eliminate or mitigate the effects of the illness.

The study limitation was the lack of participation from some patients, especially women. If all invited patients had participated, new insights regarding the complications of the disease, for example, the genitals or sexual function of women, might have been identified. There is a possibility of recall bias due to the study of surgical complications conducted after 1 year. While qualitative studies typically utilize convenient and subsequently purposive sampling, this approach can lead to sampling bias if not all patients choose to participate.

## 5 Conclusion

This study showed that, in addition to common PhCs such as pain and bleeding, there are numerous PsCs and SCs faced by patients with a history of hemorrhoidectomy. All these complications must be clearly and objectively communicated to the patient before they decide to undergo this surgery, and doctors take special measures to manage these complications properly. This disease, in addition to introducing a comprehensive and sometimes new range of PhCs related to hemorrhoidectomy, has, for the first time, successfully identified the PsCs and SCs of the disease. Researchers hope that the results of this study and the practical suggestions provided can help properly manage these complications and consequently improve the health and satisfaction of patients.

## Author Contributions

**Masoumeh Ebrahimi Tavani:** conceptualization, methodology, validation, formal analysis, supervision, writing – review and editing. **Yegane Partovi:** conceptualization, methodology, data curation, formal analysis, writing – original draft. **Tahmineh Poursaki:** conceptualization, methodology, investigation, visualization. **Farid Gharibi:** writing – review and editing, conceptualization, methodology, investigation, data curation, formal analysis, project administration.

## Conflicts of Interest

The authors declare no conflicts of interest.

## Transparency Statement

The lead author Farid Gharibi affirms that this manuscript is an honest, accurate, and transparent account of the study being reported; that no important aspects of the study have been omitted; and that any discrepancies from the study as planned (and, if relevant, registered) have been explained.

## Supporting information

Appendix‐_Interview_Checklist.

## Data Availability

The data that support the findings of this study are available from the corresponding author upon reasonable request. Data are stored at the origin of the university of study. If any researcher is interested, for a valid reason, they may contact the corresponding author (Farid Gharibi), gharibihsa@gmail.com. “All authors have read and approved the final version of the manuscript. Farid Gharibi, as the corresponding author, had full access to all of the data in this study and took complete responsibility for the integrity of the data and the accuracy of the data analysis.” “Farid Gharibi affirms that this manuscript is an honest, accurate, and transparent account of the study being reported; that no important aspects of the study have been omitted; and that any discrepancies from the study as planned (and, if relevant, registered) have been explained.”
