# Supplementary material for: The Complications of Hemorrhoidectomy From Patients' Perspective: A Qualitative Study
Source: Health Sci Rep. 2025 Apr 28;8(5):e70724. doi: 10.1002/hsr2.70724 (PMC12037693; doi:10.1002/hsr2.70724)
Supplement: Supplementary file 1 — Appendix‐_Interview_Checklist. [file HSR2-8-e70724-s001.docx]

**Interview Checklist**

- Please explain your experience with hemorrhoidal disease and the surgery you had, and what has happened to you since then?

- What physical complications did you experience after surgery?

- What psychological complications did you experience after the surgery?

- What social complications did you experience after the surgery?

- Were your social roles disrupted?
